# Effects of residential acaricide treatments on patterns of pathogen coinfection in blacklegged ticks

**DOI:** 10.1017/S0031182024000349

**Published:** 2024-08

**Authors:** Richard S. Ostfeld, Sahar Adish, Stacy Mowry, William Bremer, Shannon Duerr, Andrew S. Evans, Ilya R. Fischhoff, Fiona Keating, Jennifer Pendleton, Ashley Pfister, Marissa Teator, Felicia Keesing

**Affiliations:** 1 Cary Institute of Ecosystem Studies, Millbrook, NY 12545, USA; 2 Department of Behavioral and Community Health, Dutchess County, NY 12601, USA; 3 Bard College, Annandale, NY 12504, USA

**Keywords:** anaplasmosis, babesiosis, coinfection, co-transmission, Lyme disease, tick control, tick-borne pathogens

## Abstract

Medically important ixodid ticks often carry multiple pathogens, with individual ticks frequently coinfected and capable of transmitting multiple infections to hosts, including humans. Acquisition of multiple zoonotic pathogens by immature blacklegged ticks (*Ixodes scapularis*) is facilitated when they feed on small mammals, which are the most competent reservoir hosts for *Anaplasma phagocytophilum* (which causes anaplasmosis in humans), *Babesia microti* (babesiosis) and *Borrelia burgdorferi* (Lyme disease). Here, we used data from a large-scale, long-term experiment to ask whether patterns of single and multiple infections in questing nymphal *I. scapularis* ticks from residential neighbourhoods differed from those predicted by independent assortment of pathogens, and whether patterns of coinfection were affected by residential application of commercial acaricidal products. Quantitative polymerase chain reaction was used for pathogen detection in multiplex reactions. In control neighbourhoods and those treated with a fungus-based biopesticide deployed against host-seeking ticks (Met52), ticks having only single infections of either *B. microti* or *B. burgdorferi* were significantly less common than expected, whereas coinfections with these 2 pathogens were significantly more common. However, use of tick control system bait boxes, which kill ticks attempting to feed on small mammals, eliminated the bias towards coinfection. Although aimed at reducing the abundance of host-seeking ticks, control methods directed at ticks attached to small mammals may influence human exposure to coinfected ticks and the probability of exposure to multiple tick-borne infections.

## Graphical abstract

**Figure:**
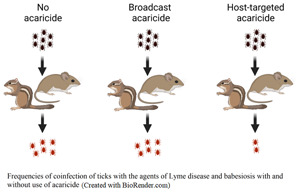

## Introduction

The use of next-generation sequencing and other molecular detection methods has revealed the presence of complex communities of microbes within individual arthropod vectors of medical and veterinary importance. If vectors are infected with multiple potentially pathogenic microbes, then their vertebrate hosts might become exposed to >1 pathogen from a single bite from that vector, altering transmission dynamics. Such a possibility can alter traditional approaches to both medicine and disease ecology. Determining the ecological causes and health consequences of exposures to a specific pathogen may require explicit information on the presence of other co-occurring pathogens (Telfer *et al*., 2010). Indeed, the expectation that vectors contain ‘pathobiomes’ suggests a new paradigm to replace the ‘one pathogen-one disease’ vision (Vayssier-Taussat *et al*., 2015). As transmission dynamics of any given vector-borne pathogen might be affected by the linked dynamics of other pathogens co-occurring with the focal pathogen within hosts and vectors (Diuk-Wasser *et al*., 2015).

Blacklegged ticks (*Ixodes scapularis*) are vectors of multiple zoonotic pathogens throughout North America. The causative agents of Lyme disease (*Borrelia burgdorferi*), human babesiosis (*Babesia microti*) and granulocytic anaplasmosis (*Anaplasma phagocytophilum*) in eastern and central North America are all transmitted predominantly by this tick vector (Eisen and Eisen, 2018). All 3 of these diseases are increasing in prevalence and pose a mounting threat to public health in North America, as well as in Europe, and parts of Asia where they are vectored by related tick species (Rochlin and Toledo, 2020). Diagnosis and treatment of infected individuals can be complicated by the simultaneous presence of >1 of these pathogens, which can alter the presentation of symptoms, increase their severity, and change the recommended therapeutic response. The presence of coinfections of individual patients by more than 1 of these pathogens is increasingly being recognized as a public health challenge (Diuk-Wasser *et al*., 2015).

Coinfections in human patients can be caused by successive bites from different ticks, each delivering only 1 species of pathogen, or by the bite of 1 coinfected tick. Widespread detection of coinfection in individual nymph-stage blacklegged ticks, which is the stage most strongly linked to human cases of tick-borne infections (Ostfeld *et al*., 2006; Pepin *et al*., 2012), suggests that the latter happens frequently. Coinfection of blacklegged ticks by *B. burgdorferi* and *B. microti* is the coinfection most commonly detected, although other pairwise combinations and coinfection with all 3 of these pathogens also occur (Lehane *et al*., 2021). Consequently, coinfected ticks pose a particularly strong threat to public health and a challenge to disease ecologists.

Of these 3 zoonotic pathogens, prevalence of *B. burgdorferi* in both ticks and human patients is generally the highest and most geographically widespread, although all of them are spreading geographically and increasing disease incidence (Eisen and Eisen, 2018). Patterns of co-occurrence of *B. burgdorferi* and *B. microti* in both reservoir hosts (Dunn *et al*., 2014; Tufts *et al*., 2023) and blacklegged ticks (Little and Molaei, 2020; Pokutnaya *et al*., 2020; Zembsch *et al*., 2021) suggest positive interactions between the 2 pathogens (i.e. facilitation), although interactions appear complex. For instance, host-to-tick transmission of *B. microti* increased when the host was also infected with *B. burgdorferi* (Dunn *et al*., 2014). Delivery to white-footed mice (*Peromyscus leucopus*) of an oral vaccine that induces immunity to *B. burgdorferi* (but not to *B. microti*) (Richer *et al*., 2014) reduced the odds of tick coinfection (*B. burgdorferi* and *B. microti*) by a factor of 7.5 despite having no direct effect on infection prevalence with the latter (Vannier *et al*., 2023). Hersh *et al*. (2012) and Keesing *et al*. (2012) demonstrated that 3 small mammal species, the white-footed mouse, the eastern chipmunk *Tamias striatus* and the short-tailed shrew *Blarina brevicauda*, were the most competent reservoir hosts for *B. microti* and *A. phagocytophilum*. These same 3 small mammals are also the most competent reservoir hosts for *B. burgdorferi* (LoGiudice *et al*., 2003, 2008; Keesing *et al*., 2009). Hersh *et al*. (2014) determined that coinfection of nymphal blacklegged ticks with *B. burgdorferi* and *B. microti* was significantly more frequent than expected from a null model that assumed independent transmission by each pathogen, suggesting that these 2 pathogens were co-transmitted from hosts to ticks. Supporting that hypothesis, Hersh *et al*. (2014) found that the great majority of coinfected ticks had fed from 1 of the 3 species of small mammal hosts.

The Tick Project (Keesing *et al*., 2022) was designed to assess the efficacy of 2 tick-killing interventions in reducing abundance of blacklegged ticks, encounters with ticks and cases of tick-borne disease. One of the acaricidal interventions was a fungal biocontrol agent (Met52), consisting of spores of the entomopathogenic fungus *Metarhizium brunneum*, sprayed on low vegetation where host-seeking ticks are likely to occur. The other was a host-targeted acaricide deployed in devices (tick control system [TCS] bait boxes) that allowed small mammals to self-apply the acaricide fipronil, but excluded other hosts, thus targeting ticks that occur on mice, chipmunks and shrews. Placebo controls for both these interventions were also deployed. We predicted that nymphal blacklegged ticks in areas treated with placebo controls would show coinfection rates of *B. burgdorferi* and *B. microti* that were significantly more frequent than expected assuming independent host-to-tick transmission, reflecting results of Hersh *et al*. (2014). We further predicted that the use of acaricide targeted at ticks on small mammals would selectively reduce survival of the larval blacklegged ticks most likely to acquire infection with both pathogens. Survival of larval ticks feeding on other, larger hosts, such as raccoons (*Procyon lotor*), opossums (*Didelphis virginiana*) and white-tailed deer (*Odocoileus virginianus*), would not be affected by this treatment, and these non-small-mammal hosts do not contribute to coinfection (Hersh *et al*., 2014). Therefore, we expected that the TCS bait boxes would eliminate the bias towards coinfection at the subsequent nymphal stage. If these predictions were supported, we expected that the use of acaricides targeted at small-mammal hosts could, in addition to reducing overall tick abundance, have the added benefit of reducing the probability of human exposure to multiple pathogens given a single tick bite.

## Materials and methods

We collected data for this research as part of The Tick Project, a multi-year study to test the effects of environmental interventions on tick abundance and infection, as well as tick-borne diseases of humans and outdoor pets, in 24 residential neighbourhoods of Dutchess County, New York, USA (Keesing *et al*., 2022; Ostfeld *et al*., 2023*a*, 2023*b*), an area of very high endemicity for multiple tick-borne diseases (Keesing *et al*., 2022). In The Tick Project, we tested the effects of 2 commercially available products, Met52 and TCS bait boxes. Met52 contains spores derived from a naturally occurring fungus, *M. brunneum*, and is applied to habitats such as lawns and gardens with a high-pressure sprayer. Application was at 175–200 psi (pounds per square inch) and conformed to product labelling. This product is intended to kill all life stages of ticks while they are seeking hosts. The second product, the TCS bait box, consists of a small plastic and metal box (19.05 cm by 13.97 cm by 6.35 cm) that contains a small amount of bait. The bait attracts small mammals like rodents and shrews (Ostfeld, 2012), which are dabbed with an acaricide, fipronil, while they are inside the box, and then exit the box unharmed. The acaricide kills ticks for several weeks after application, with the goal of reducing transmission of tick-borne pathogens. Details of these treatments and other aspects of the study design are provided in Keesing *et al*. (2022).

Each of the 24 neighbourhoods in The Tick Project was randomly assigned to receive 1 of 4 possible combinations of the 2 products – Met52 and TCS bait boxes – or their equivalent placebo controls. Placebo controls consisted of the product without its active ingredient. The placebo for Met52 was a high-pressure spray of water without the fungus. The placebo for TCS bait boxes was the box with the bait but without the acaricide. Thus, there were 4 treatment combinations – with active Met52 and active TCS bait boxes, with active Met52 and placebo bait boxes, with placebo Met52 and active TCS bait boxes and with placebos of both treatments. The 24 neighbourhoods were randomly grouped into 6 replicates of each of the 4 treatment combinations. The study was thus randomized, replicated and placebo-controlled. It was also double-masked (i.e. double-blinded) as neither the participants in the neighbourhoods nor anyone collecting data for the project knew the treatment assignments of any of the neighbourhoods.

Residents were invited to participate in the project if they lived in 1 of the 24 neighbourhoods, each of which consisted of ~100 homes and had a history of high incidence of tick-borne illnesses (Keesing *et al*., 2022). After thorough canvasing to determine interest and eligibility (e.g. willingness to forgo other acaricidal treatments for the duration of the study), we enrolled 24–44% (mean 34%) of households in each neighbourhood.

We collected questing nymphal ticks on the properties of 20 randomly selected households twice per year in each neighbourhood in May–July of 2017, 2018, 2019 and 2021, at the peak of nymphal activity for blacklegged ticks. (We were not able to collect ticks in 2020 because of the COVID-19 pandemic.) Ticks were collected by flag-sampling in 3 common habitat types in residential areas of Dutchess County, lawns, gardens and forests. Flag-sampling was standardized among all researchers by total effort (flagging time) and effort per habitat type (proportional to habitat area). All collected nymphal ticks from all 3 habitats were pooled by neighbourhood and stored alive in humidified vials until being flash-frozen and stored (see below).

We tested collected nymphal blacklegged ticks for the presence of 3 tick-borne pathogens, *B. burgdorferi* (causative agent of Lyme disease), *A. phagocytophilum* (anaplasmosis) and *B. microti* (babesiosis). Details of this procedure are provided in Ostfeld *et al*. (2023*b*). Briefly, we surface-sterilized ticks with 10% bleach within 2–3 weeks of collection, and then rinsed them with deionized water, after which they were stored individually in 2 mL Eppendorf tubes at −80°C. After lysing *via* bead-beating and homogenizing the ticks, we extracted DNA using the DNeasy 96 Blood & Tissue kit (Qiagen, Maryland, USA). We used multiplex polymerase chain reaction(PCR) to detect *A. phagocytophilum* and *B. burgdorferi*, as described in Ostfeld *et al*. (2023*b*). Briefly, *A. phagocytophilum* was detected by targeting the msp2 gene using primers ApMSP2f, ApMSP2r and ApMSP2p (Courtney *et al*., 2004; Keesing *et al*., 2012). *Borrelia burgdorferi* was detected by targeting the 23S ribosomal RNA (rRNA) gene of *B. burgdorferi* using primers Bb23Sf, Bb23Sr and Bb23Sp. *Babesia microti* was detected by targeting the 18S rRNA gene using primers smbaJF and smbaKR following a melting curve analysis (Ostfeld *et al*., 2023*b*).

We ran all reactions on a LightCycler 480 II (Roche, Switzerland) thermal cycler following manufacturer recommendations, and used DNA extract from tick larvae and PCR-grade water (Roche) as negative controls (Ostfeld *et al*., 2023*b*). Positive controls were taken from previous positive control samples (Hersh *et al*., 2012; Keesing *et al*., 2012). We tested 3 replicates of each nymphal tick sample, and assigned infection status based on the details provided in Ostfeld *et al*. (2023*b*). For analysis, we considered nymphal ticks collected in 2018–2021 for which we were able to determine infection status for all 3 pathogens. The nymphal ticks collected these years represent the cohorts that could have been affected by the acaricidal interventions, which began in 2017.

We determined whether nymphal ticks in each treatment were coinfected more or less than expected by chance using a permutation test developed by Hersh *et al*. (2014). First, we combined infection status for the nymphal ticks from each of the 4 treatment combinations, so that we had 4 sets of data. For each treatment combination, we randomly resampled nymphal tick infection status for each pathogen, independently and without replacement, 100 000 times. This allowed us to determine the expected frequencies of each pathogen alone and in combination with other pathogens, which we then compared to the observed frequencies of single and multiple infections from that treatment combination. To do this, we determined the proportion of samples in which the difference between the observed prevalence of each infection type and the permutation mean was as or more extreme than the difference between the permutation mean and each permuted sample. The statistical significance of this result was determined as *P*  =  (number of samples in which [permutation mean – observed data] ⩾ [permutation mean – permutation data point] + 1)/(number of permutations + 1) (Chihara and Hesterberg, 2011; Hersh *et al*., 2014). We quantified the effect size by determining the ratio of the observed level of co-infection to the permutation mean.

To determine whether there were effects of treatment on observed levels of coinfection, we first excluded neighbourhood–year combinations in which we did not collect at least 10 ticks, of which there were 7 occurrences out of 72 total. We used generalized linear mixed models (glmm), with an interaction between the 2 treatments (Baitbox × Met52) and year (2018, 2019, 2021) as fixed effects, and neighbourhood as a random effect. For these analyses, we had 6 replicates (i.e. neighbourhoods) for each of the 4 treatments, and we used the number of infected ticks in a neighbourhood each year as the dependent variable, with an offset for the total number of ticks collected from that neighbourhood. Models were fitted with a negative binomial distribution. We tested that data met assumptions of tests using package *DHARMa* (Hartig, 2022), and determined statistical significance using the function *Anova* from package *car* (Fox and Weisberg, 2019). Data were analysed using version 4.0.1 of R.

## Results

We collected and tested 5231 questing nymphal blacklegged (*I. scapularis*) ticks for the presence of all 3 pathogens. *Borrelia burgdorferi* had the highest prevalence, infecting an overall mean of 21% (±0.02% s.e.m.) of ticks in the control neighbourhoods (i.e. those neighbourhoods treated only with placebo interventions). In contrast, 13% (±0.03%) of nymphs in control neighbourhoods were infected with *A. phagocytophilum*, and 9% (±0.01%) with *B. microti*.

Compared to single infections, coinfections were relatively rare, with a mean of 4% (±0.01%) of ticks in control neighbourhoods infected with both *B. burgdorferi* and *B. microti*, the most common coinfection. An overall mean of 2% (±0.01%) of nymphal ticks were infected with both *B. burgdorferi* and *A. phagocytophilum*, and 1% (±0.004%) with *A. phagocytophilum* and *B. microti*. Ticks infected with all 3 pathogens were uncommon, accounting for <1% of ticks in control neighbourhoods.

**Figure 1.**
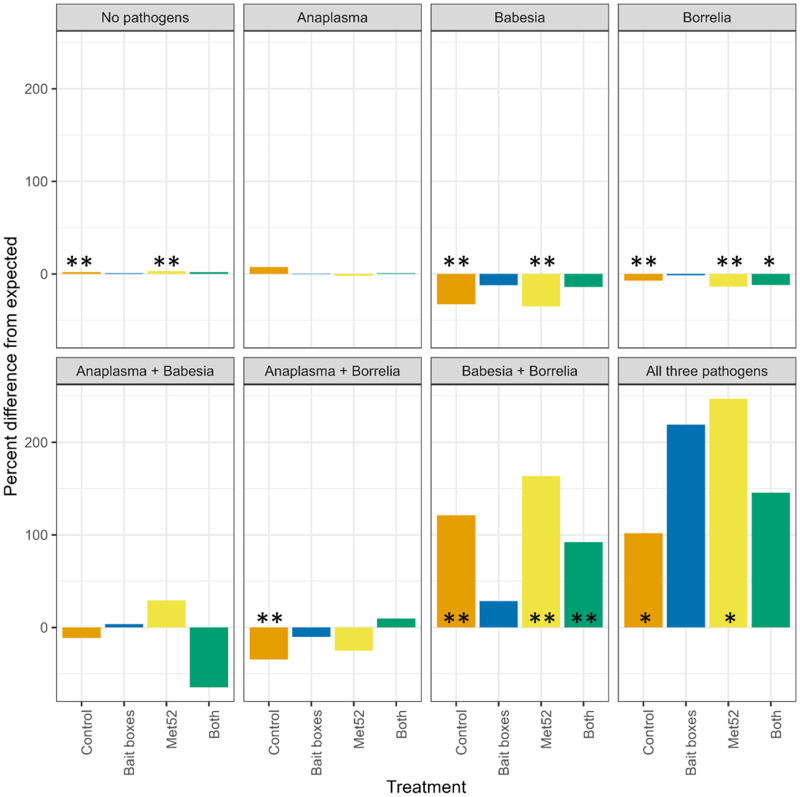
Per cent difference in observed prevalence of questing nymphal blacklegged ticks from values expected if prevalence of a particular pathogen in ticks were random with respect to that of other pathogens. Zero values indicate that observed prevalences were equal to expected. Control neighbourhoods were untreated, ‘both’ indicates neighbourhoods treated with bait boxes and Met52 spray, see details in *Methods*. Asterisks indicate statistically significant differences, * indicates *P* < 0.05, and ** indicates *P* < 0.01. See also Table S1 and Fig. S1.

Based on our permutation analysis, ticks in control neighbourhoods were significantly less likely to be infected with only *B. burgdorferi* or only *B. microti* than expected from the assumption of independent transmission of each pathogen. For example, if infection were random, 6% (95% CI 5.30–6.64) of ticks on control plots would be infected with just *B. microti*, but we found that only 4% were, which is 2/3 of the expected value (Table S1; Fig. 1; Additional file 1: Fig. S1).

The permutation analysis also revealed that some coinfections in control neighbourhoods were more common than expected from the assumption of independent transmission of each pathogen. In particular, ticks infected with *B. burgdorferi* were significantly more likely than expected to also be infected with *A. phagocytophilum* (*P* = 0.010), *B. microti* (*P* < <0.01) or both (*P* < 0.05) (Table S1; Fig. 1).

Neighbourhoods treated with active Met52 fungal spray showed patterns of infection and coinfection similar to those seen in control neighbourhoods (Table S1; Fig. 1). However, in sharp contrast to the patterns observed in control neighbourhoods, none of the observed levels of multiple infections in neighbourhoods treated with active bait boxes were significantly different from those expected by chance (Table S1; Fig. 1). In neighbourhoods treated with both bait boxes and Met52, *B. burgdorferi* was significantly less common than expected as a single infection, and significantly more common as a coinfection with *B. microti*.

**Figure 2.**
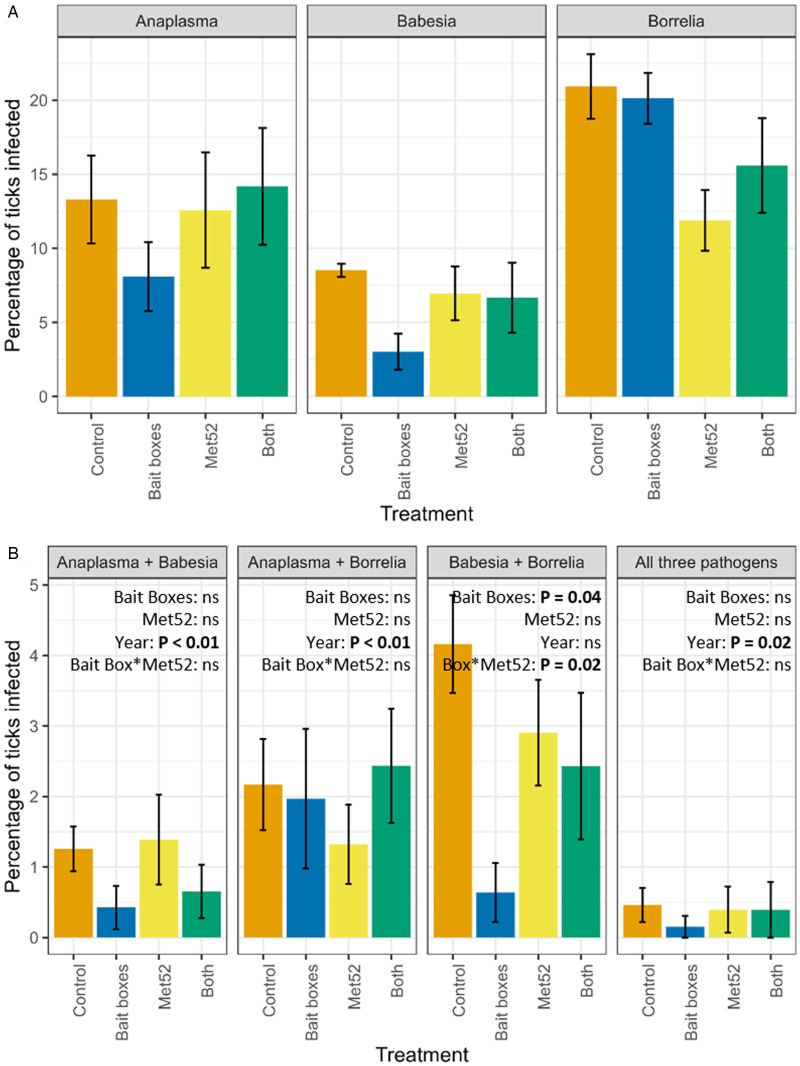
Mean (±standard error of the mean) percentage of questing nymphal blacklegged ticks infected with (A) individual pathogens, and (B) multiple pathogens in neighbourhoods in each of the 4 treatments of the Tick Project. Data on individual pathogens include ticks that were coinfected, and data on double infections include ticks that were triply infected. For example, the percentage of ticks infected with *Anaplasma phagocytophilum* in (A) includes ticks that were also infected with other pathogens, as in (B). Control neighbourhoods were untreated, ‘both’ indicates neighbourhoods treated with bait boxes and Met52 spray, see details in *Methods*. Effects of treatments on individual pathogens were previously reported in Ostfeld *et al*. (2023*a*, 2023*b*) and are included here for reference. Note that *y*-axis values vary.

Using the 6 neighbourhoods receiving each treatment as replicates, we asked whether treatment significantly affected the proportion of ticks coinfected with each combination of pathogens. In the neighbourhoods treated with bait boxes, we observed a statistically significant reduction in the proportion of ticks coinfected with both *B. burgdorferi* and *B. microti*, as compared to neighbourhoods treated with placebo controls (*P* = 0.04; Fig. 2B). There was also a significant interaction between bait boxes and Met52 spray (*P* = 0.03; Fig. 2B). None of the other combinations of coinfection showed a significant effect of treatment. In contrast, 3 of the 4 possible coinfections showed statistically significant effects of year (Fig. 2; Additional file 2: Fig. S2), with the prevalence of coinfections generally declining over time in all treatments, including the controls.

## Discussion

We tested 5231 questing nymphal *I. scapularis* ticks collected over 4 years in Dutchess County, NY, to estimate the prevalence of single infections and 2- and 3-way coinfections with the most frequently encountered tick-borne, zoonotic pathogens in eastern North America. Assessments of ticks collected from control neighbourhoods, in which no acaricidal treatments were deployed, confirmed that ticks coinfected with *B. burgdorferi* and either *B. microti* or *A. phagocytophilum* occurred more frequently than expected assuming independent transmission (Hersh *et al*., 2014). In parallel, ticks infected with only 1 of these pathogens occurred less frequently than expected under independent transmission, in the control neighbourhoods. Prior research revealed that blacklegged ticks acquire coinfections of tick-borne pathogens predominantly by feeding as larvae on white-footed mice, eastern chipmunks or short-tailed shrews (Hersh *et al*., 2014), because these hosts are themselves often infected with multiple pathogens and are more efficient than other vertebrate hosts at transmitting infections to ticks. This observation led to the hypothesis that the selective killing of ticks that feed as larvae on this guild of small-mammal hosts would reduce coinfection prevalence within host-seeking ticks (because the remaining nymphs would have fed in higher numbers on other hosts), in addition to reducing tick abundance (which is the intended effect).

Supporting the hypothesis that selectively killing ticks that feed on small mammals would disrupt transmission biased towards multiple pathogens, we found that the questing nymphs collected from neighbourhoods in which TCS bait boxes were deployed showed coinfection patterns that were indistinguishable from expectations based on independent transmission. Prevalence of single infections with each of the 3 pathogens under bait box treatments was not different from expectations arising from the assumption of independent transmission. In other words, the bias towards coinfection that occurs under unmanipulated conditions (Hersh *et al*., 2014) was eliminated by the use of bait boxes. The selective killing of ticks feeding as larvae on small mammals (Dolan *et al*., 2004; Stafford *et al*., 2017; Williams *et al*., 2018*a*, 2018*b*) eliminated the observed bias towards coinfection (Hersh *et al*., 2014; Little and Molaei, 2020; Zembsch *et al*., 2021; but see Little *et al*., 2020) and away from single infection that arises because mice, chipmunks and shrews are the most competent reservoirs for all 3 of the zoonotic pathogens under study (Levi *et al*., 2016). We surmise that the questing nymphal ticks we collected from the neighbourhoods with active bait boxes had fed as larvae largely on other, non-small-mammal members of the host community, which would not result in a bias towards co-occurrence of multiple pathogens in individual nymphal ticks. Indeed, in neighbourhoods treated with Met52, which affects host-seeking ticks unselectively, the bias towards coinfection observed in the controls was maintained, as expected. Not surprisingly, ticks collected from neighbourhoods with both bait boxes and Met52 showed intermediate patterns of single- and coinfection.

Our prior analyses of the effects of both Met52 and TCS bait boxes on prevalence of tick infection with zoonotic pathogens focused exclusively on single, rather than multiple, infections (Ostfeld *et al*., 2023*b*). Those analyses revealed no significant effects of TCS bait boxes on infection prevalence of nymphal ticks with any of the 3 single pathogens but a significantly reduced infection prevalence with *B. burgdorferi* (but not of *B. microti* or *A. phagocytophilum*) in neighbourhoods with active Met52. These results caused us to expect that coinfections of *B. burgdorferi* and either of the other 2 pathogens would be reduced in the Met52-treated neighbourhoods, all else equal, but this hypothesis was not supported.

The use of bait boxes significantly reduced coinfection of *B. burgdorferi* and *B. microti* but not of *B. burgdorferi* and *A. phagocytophilum*. A reduced effect of bait boxes on the latter coinfection might arise because small mammals are only modestly more competent reservoirs for *A. phagocytophilum* as compared to other vertebrate hosts (Keesing *et al*., 2012). For *B. microti*, the difference in reservoir competence between small mammals and other hosts is more distinct (Hersh *et al*., 2012). Thus, targeting ticks that fed as larvae on small mammals might be expected to have a weaker effect on coinfections involving *A. phagocytophilum*. The potential roles of indirect, host-mediated interactions between *B. burgdorferi* and the other 2 pathogens (Tufts *et al*., 2023; Vannier *et al*., 2023), and of vertical transmission of *B. microti* (Tufts and Diuk-Wasser, 2018), are not clear but are worthy of further study.

Reports of human patients concurrently infected with *B. burgdorferi* and *B. microti* have accumulated since the 1990s, with evidence for potentially increased severity and duration of symptoms (Krause *et al*., 1996, 2002; Caulfield and Pritt, 2015), but see Wormser *et al*. (2019). Coinfections in human patients can arise from multiple tick bites, each resulting in single infections, or from bites from coinfected ticks, with the relative frequency of these 2 modes of transmission unknown, to our knowledge. Means of preventing such coinfections could protect public health by avoiding difficulties involved with diagnosing and treating coinfections within individuals. Although acaricides are expected to aid in preventing exposures by reducing tick abundance (Dolan *et al*., 2004; Schulze *et al*., 2007, 2017; Williams *et al*., 2018*a*, 2018*b*; Jordan and Schulze, 2019; Little *et al*., 2020; but see Hinckley *et al*., 2016; Hinckley *et al*., 2021; Keesing *et al*., 2022), an under-recognized pathway for acaricides directed at small-mammal hosts might consist of reducing the bias towards coinfections in host-seeking nymphal ticks.

## Supporting information

Ostfeld et al. supplementary material 1 Ostfeld et al. supplementary material

Ostfeld et al. supplementary material 2 Ostfeld et al. supplementary material

Ostfeld et al. supplementary material 3 Ostfeld et al. supplementary material

Ostfeld et al. supplementary material 4 Ostfeld et al. supplementary material

Ostfeld et al. supplementary material 5 Ostfeld et al. supplementary material

## Data Availability

Data will be posted on the Cary Institute's Figshare site upon acceptance of the final manuscript.

## Supplementary material

The supplementary material for this article can be found at https://doi.org/10.1017/S0031182024000349

## References

[ref1] Caulfield AJ and Pritt BS (2015) Lyme disease coinfections in the United States. Clinics in Laboratory Medicine 35, 827–846. 26593260 10.1016/j.cll.2015.07.006

[ref2] Chihara L and Hesterberg T (2011) Mathematical Statistics with Resampling and R. Hoboken, NJ: Wiley.

[ref3] Courtney JW, Kostelnik LM, Zeidner NS and Massung RF (2004) Multiplex real-time PCR for detection of *Anaplasma phagocytophilum* and *Borrelia burgdorferi*. Journal of Clinical Microbiology 42, 3164–3168. 15243077 10.1128/JCM.42.7.3164-3168.2004 PMC446246

[ref4] Diuk-Wasser M, Vannier E and Krause PJ (2015) Coinfection by *Ixodes* tick-borne pathogens: ecological epidemiological, and clinical consequences. Trends in Parasitology 32, 30–42. 26613664 10.1016/j.pt.2015.09.008 PMC4713283

[ref5] Dolan MC, Maupin GO, Schneider BS, Denatale C, Hamon N and Cole C (2004) Control of immature *Ixodes scapularis* (Acari: Ixodidae) on rodent reservoirs of *Borrelia burgdorferi* in a residential community of Southeastern Connecticut. Journal of Medical Entomology 41, 1043–1054. 15605643 10.1603/0022-2585-41.6.1043

[ref6] Dunn JM, Krause PJ, Davis S, Vannier EG, Fitzpatrick MC, Rollend L, Belperron AA, States SL, Stacey A, Bockenstedt LK, Fish D and Diuk-Wasser M (2014) *Borrelia burgdorferi* promotes the establishment of *Babesia microti* in the northeastern United States. PLoS ONE 9, e115494. 25545393 10.1371/journal.pone.0115494 PMC4278703

[ref7] Eisen RJ and Eisen L (2018) The blacklegged tick, *Ixodes scapularis*: an increasing public health concern. Trends in Parasitology 34, 295–309. 29336985 10.1016/j.pt.2017.12.006 PMC5879012

[ref8] Fox J and Weisberg S (2019) An R Companion to Applied Regression, 3rd Edn. Thousand Oaks, CA: Sage Publications. Available at https://socialsciences.mcmaster.ca/jfox/Books/Companion/

[ref9] Hartig F (2022) DHARMa: Residual diagnostics for hierarchical (multi-level/mixed) regression models_. R package version 0.4.6. Available at https://CRAN.R-project.org/package=DHARMa

[ref10] Hersh M, Tibbetts M, Ostfeld RS, Straus M and Keesing F (2012) Quantifying reservoir competence for *Babesia microti* of wildlife host species using real-time PCR. Emerging Infectious Diseases 18, 1951–1957. 23171673 10.3201/eid1812.111392 PMC3557901

[ref11] Hersh MH, Ostfeld RS, McHenry DJ, Tibbetts M, Brunner JL, Killilea ME, LoGiudice K, Schmidt KA and Keesing F (2014) Co-Infection of blacklegged ticks with *Babesia microti* and *Borrelia burgdorferi* is higher than expected and acquired from small mammal hosts. PLoS ONE 9, e99348. 24940999 10.1371/journal.pone.0099348 PMC4062422

[ref12] Hinckley AF, Meek JI, Ray JAE, Niesobecki SA, Connally NP and Feldman KA (2016) Effectiveness of residential acaricides to prevent Lyme and other tick-borne diseases in humans. The Journal of Infectious Diseases 214, 182–188. 26740276 10.1093/infdis/jiv775 PMC10874626

[ref13] Hinckley AF, Niesobecki SA, Connally NP, Hook SA, Biggerstaff BJ and Horiuchi KA (2021) Prevention of Lyme and other tickborne diseases using a rodent-targeted approach: a randomized controlled trial in Connecticut. Zoonoses and Public Health 68, 578–587. 34050628 10.1111/zph.12844 PMC10898493

[ref14] Jordan RA and Schulze TL (2019) Ability of two commercially available host-targeted technologies to reduce abundance of *Ixodes scapularis* (Acari: Ixodidae) in a residential landscape. Journal of Medical Entomology 56, 1095–1101. 30984975 10.1093/jme/tjz046 PMC8116133

[ref15] Keesing F, Brunner J, Duerr S, Killilea M, LoGiudice K, Schmidt K, Vuong H and Ostfeld RS (2009) Hosts as ecological traps for the vector of Lyme disease. Proceedings of the Royal Society B: Biological Sciences 276, 3911–3919. 10.1098/rspb.2009.1159 PMC2825780 19692412

[ref16] Keesing F, Hersh MH, Tibbetts M, McHenry DJ, Duerr S, Brunner J, Killilea M, LoGiudice K, Schmidt KA and Ostfeld RS (2012) Reservoir competence of vertebrate hosts for *Anaplasma phagocytophilum*. Emerging Infectious Diseases 18, 2013–2016. 23171835 10.3201/eid1812.120919 PMC3557888

[ref17] Keesing F, Mowry S, Bremer W, Duerr S, Evans AS, Fischhoff IR, Hinckley AF, Hook SA, Keating F, Pendleton J, Pfister A, Teator M and Ostfeld RS (2022) Effects of tick-control interventions on tick abundance, human encounters with ticks, and incidence of tickborne diseases in residential neighbourhoods, New York, USA. Emerging Infectious Diseases 28, 957–966. 35447066 10.3201/eid2805.211146 PMC9045441

[ref18] Krause PJ, Telford SR, Spielman A, Sikand V, Ryan R, Christianson D, Burke G, Brassard P, Pollack R, Peck J and Persing DH (1996) Concurrent Lyme disease and babesiosis: evidence for increased severity and duration of illness. Journal of the American Medical Association 275, 1657–1660. 8637139

[ref19] Krause PJ, McKay K, Thompson CA, Sikand VK, Lentz R and Lepore T (2002) Disease-specific diagnosis of coinfecting tickborne zoonoses: babesiosis human granulocytic ehrlichiosis, and Lyme disease. Clinical Infectious Diseases 34, 1184–1191. 11941544 10.1086/339813

[ref20] Lehane A, Maes SE, Graham CB, Jones E, Delorey M and Eisen RJ (2021) Prevalence of single and coinfections of human pathogens in Ixodes ticks in the United States, 2013-2019. Ticks and Tick-borne Diseases 12, 101637. 33360805 10.1016/j.ttbdis.2020.101637 PMC11351056

[ref21] Levi T, Keesing F, Holt RD, Barfield M and Ostfeld RS (2016) Quantifying dilution and amplification in a community of hosts for tick-borne pathogens. Ecological Applications 26, 484–498. 27209790 10.1890/15-0122

[ref22] Little EAH and Molaei G (2020) Passive tick surveillance: exploring spatiotemporal associations of *Borrelia burgdorferi* (Spirochaetales: Spirochaetaceae), *Babesia microti* (Piroplasmida: Babesiidae), and *Anaplasma phagocytophilum* (Rickettsiales: Anaplasmataceae) infection in *Ixodes scapularis* (Acari: Ixodidae). Vector-Borne and Zoonotic Diseases 20, 177–186. 31580216 10.1089/vbz.2019.2509

[ref23] Little EAH, Williams SC, Stafford KC, Linske MA and Molaei G (2020) Evaluating the effectiveness of an integrated tick management approach on multiple pathogen infection in *Ixodes scapularis* questing nymphs and larvae parasitizing white-footed mice. Experimental and Applied Acarology 80, 127–136. 31853763 10.1007/s10493-019-00452-7

[ref24] LoGiudice K, Ostfeld RS, Schmidt KA and Keesing F (2003) The ecology of infectious disease: effects of host diversity and community composition on Lyme disease risk. Proceedings of the National Academy of Sciences of the USA 100, 567–571. 12525705 10.1073/pnas.0233733100 PMC141036

[ref25] LoGiudice K, Duerr S, Newhouse M, Schmidt K, Killilea M and Ostfeld RS (2008) Impact of host community composition on Lyme disease risk. Ecology 89, 2841–2849. 18959321 10.1890/07-1047.1

[ref26] Ostfeld RS (2012) Lyme Disease: The Ecology of A complex System. New York, NY: Oxford University Press.

[ref27] Ostfeld RS, Price A, Hornbostel VL, Benjamin MA and Keesing F (2006) Controlling ticks and tick-borne zoonoses with biological and chemical agents. Bioscience 56, 383–394.

[ref28] Ostfeld RS, Mowry S, Bremer W, Duerr S, Evans Jr AS and Fischhoff IR (2023a) Impacts over time of neighbourhood-scale interventions to control ticks and tick-borne disease incidence. Vector-Borne and Zoonotic Diseases 23, 89–105. 36848248 10.1089/vbz.2022.0094 PMC9993163

[ref29] Ostfeld RS, Adish S, Mowry S, Bremer W, Duerr S, Evans Jr AS, Fischhoff IR, Keating F, Pendleton J, Pfister A, Teator M and Keesing F (2023b) Effects of neighbourhood-scale acaricidal treatments on infection prevalence of blacklegged ticks (*Ixodes scapularis*) with three zoonotic pathogens. Pathogens 12, 172. 36839444 10.3390/pathogens12020172 PMC9960617

[ref30] Pepin KM, Eisen RJ, Mead PS, Piesman J, Fish D, Hoen AG, Barbour AG, Hamer S and Diuk-Wasser MA (2012) Geographic variation in the relationship between human Lyme disease incidence and density of infected host-seeking *Ixodes scapularis* nymphs in the eastern United States. American Journal of Tropical Medicine and Hygiene 86, 1062–1071. 22665620 10.4269/ajtmh.2012.11-0630 PMC3366524

[ref31] Pokutnaya D, Molaei G, Weinberger DM, Vossbrinck CR and Diaz AJ (2020) Prevalence of infection and co-infection and presence of rickettsial endosymbionts in *Ixodes scapularis* (Acari: Ixodidae) in Connecticut, USA. Journal of Parasitology 106, 30–37. 31971489

[ref32] Richer LC, Brisson D, Melo R, Ostfeld RS, Zeidner N and Gomes-Solecki M (2014) Reservoir targeted vaccine against *Borrelia burgdorferi*: a new strategy to prevent Lyme disease transmission. The Journal of Infectious Diseases 209, 1972–1980. 24523510 10.1093/infdis/jiu005 PMC4038139

[ref33] Rochlin I and Toledo A (2020) Emerging tick-borne pathogens of public health importance: a mini-review. Journal of Medical Microbiology 69, 781–791. 32478654 10.1099/jmm.0.001206 PMC7451033

[ref34] Schulze TL, Jordan RA, Schulze CJ, Healy SP, Jahn MB and Piesman J (2007) Integrated use of 4-poster passive topical treatment devices for deer, targeted acaricide applications, and maxforce TMS bait boxes to rapidly suppress populations of *Ixodes scapularis* (Acari: Ixodidae) in a residential landscape. Journal of Medical Entomology 44, 830–839. 17915516 10.1603/0022-2585(2007)44[830:iuoppt]2.0.co;2

[ref35] Schulze TL, Jordan RA, Williams M and Dolan MC (2017) Evaluation of the SELECT tick control system (TCS), a host-targeted bait box, to reduce exposure to *Ixodes scapularis* (Acari: Ixodidae) in a Lyme disease endemic area of New Jersey. Journal of Medical Entomology 54, 1019–1024. 28399280 10.1093/jme/tjx044 PMC5968626

[ref36] Stafford III KC, Williams SC and Molaei G (2017) Integrated pest management in controlling ticks and tick-associated diseases. Journal of Integrated Pest Management 8, 12–28.

[ref37] Telfer S, Lambin X, Birtles R, Beldomenico P, Burthe S, Paterson S and Begon M (2010) Species interactions in a parasite community drive infection risk in a wildlife population. Science 330, 243–246. 20929776 10.1126/science.1190333 PMC3033556

[ref38] Tufts DM and Diuk-Wasser MA (2018) Transplacental transmission of tick-borne *Babesia microti* in its natural host *Peromyscus leucopus*. Parasites & Vectors 11, 1–9. 29728129 10.1186/s13071-018-2875-8 PMC5935994

[ref39] Tufts DM, Adams B and Diuk-Wasser M (2023) Ecological interactions driving population dynamics of two tick-borne pathogens, *Borrelia burgdorferi* and *Babesia microti*. Proceedings of the Royal Society B: Biological Sciences 290, 20230642. 10.1098/rspb.2023.0642 PMC10291726 37357860

[ref40] Vannier E, Richer L, Dinh D, Brisson D, Ostfeld RS and Gomes-Solecki M (2023) Deployment of a reservoir-targeted vaccine against *Borrelia burgdorferi* reduces the prevalence of *Babesia microti* coinfection in *Ixodes scapularis* ticks. The Journal of Infectious Diseases 227, 1127–1131. 36416014 10.1093/infdis/jiac462 PMC10175066

[ref41] Vayssier-Taussat M, Kaximirova M, Hubalek Z, Hornok S, Farkas R, Cosson J, Bonnet S, Vourch G, Gasqui P, Mihalca AD, Plantard O, Silaghi C, Cutler S and Rizzoli A (2015) Emerging horizons for tick-borne pathogens: from the ‘one pathogen-one disease’ vision to the pathobiome paradigm. Future Microbiology 10, 2033–2043. 26610021 10.2217/fmb.15.114 PMC4944395

[ref42] Williams SC, Little EAH, Stafford KC, Molaei G and Linske MA (2018a) Integrated control of juvenile *Ixodes scapularis* parasitizing *Peromyscus leucopus* in residential settings in Connecticut, United States. Ticks and Tick-borne Diseases 9, 1310–1316. 29859885 10.1016/j.ttbdis.2018.05.014

[ref43] Williams SC, Stafford KC, Molaei G and Linske MA (2018b) Integrated control of nymphal *Ixodes scapularis*: effectiveness of white-tailed deer reduction, the entomopathogenic fungus *Metarhizium anisopliae*, and fipronil-based rodent bait boxes. Vector-Borne and Zoonotic Diseases 18, 55–64. 29173127 10.1089/vbz.2017.2146

[ref44] Wormser GP, McKenna D, Scavarda C, Cooper D, El Khoury MY, Nowakowski J, Sudhindra P, Ladenheim A, Wang G, Karmen CL, Demarest V, Dupuis APII and Wong SJ (2019) Co-infections in persons with early Lyme disease, New York, USA. Emerging Infectious Diseases 25, 748–752. 30882316 10.3201/eid2504.181509 PMC6433014

[ref45] Zembsch TE, Lee X, Bron GM, Bartholomay LC and Paskewitz SM (2021) Coinfection of *Ixodes scapularis* (Acari: Ixodidae) nymphs with *Babesia* spp. (Piroplasmida: Babesiidae) and *Borrelia burgdorferi* Spirochaetales: Spirochaetaceae) in Wisconsin. Journal of Medical Entomology 58, 1891–1899. 33855361 10.1093/jme/tjab056

