# Supplementary figures and images for: Effects of residential acaricide treatments on patterns of pathogen coinfection in blacklegged ticks
Source: Parasitology. 2024 Mar 18;151(9):946–52. doi: 10.1017/S0031182024000349 (PMC11770522; doi:10.1017/S0031182024000349)

Expected prevalence  $\pm$  95% confidence interval

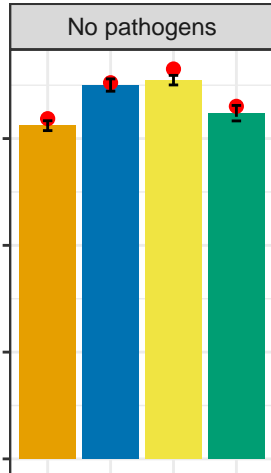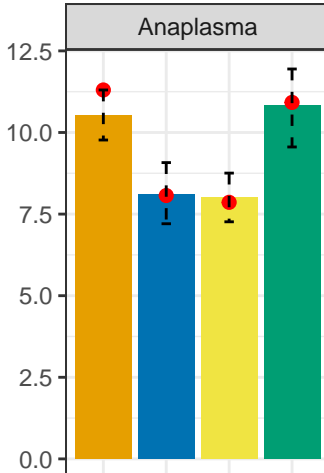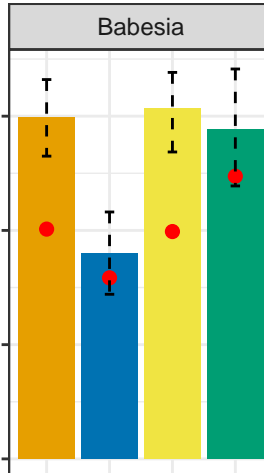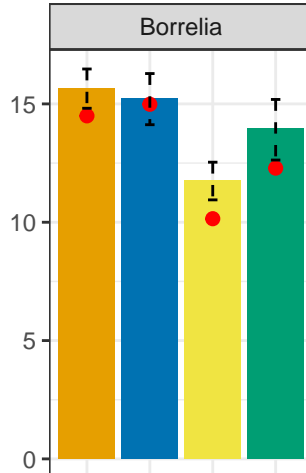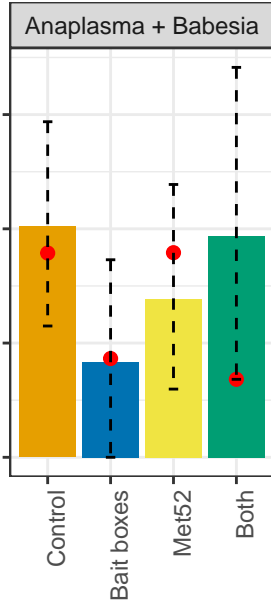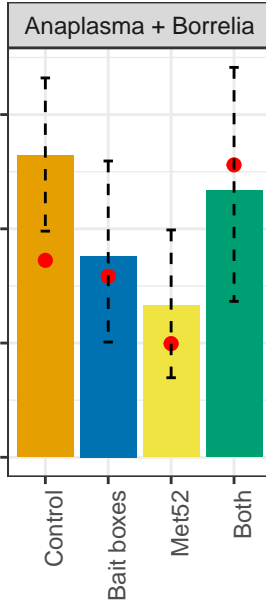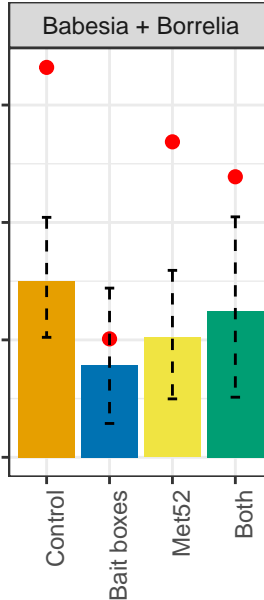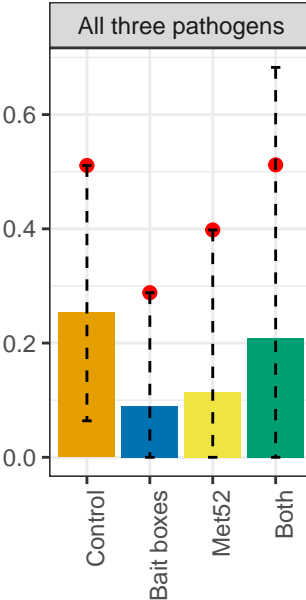

Treatment

Supplement: Ostfeld et al. supplementary material 1 — Ostfeld et al. supplementary material [file S0031182024000349sup001.pdf]

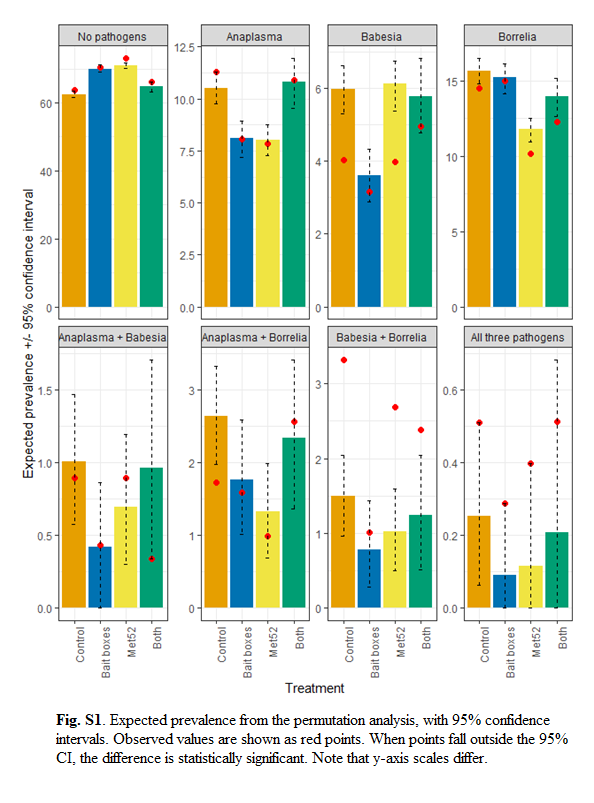

Supplement: Ostfeld et al. supplementary material 3 — Ostfeld et al. supplementary material [file S0031182024000349sup003.tif]

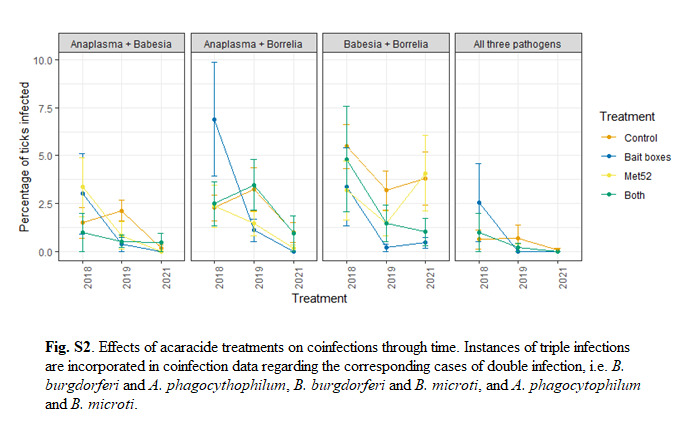

Supplement: Ostfeld et al. supplementary material 4 — Ostfeld et al. supplementary material [file S0031182024000349sup004.tif]
