# Supplementary material for: Effects of residential acaricide treatments on patterns of pathogen coinfection in blacklegged ticks
Source: Parasitology. 2024 Mar 18;151(9):946–52. doi: 10.1017/S0031182024000349 (PMC11770522; doi:10.1017/S0031182024000349)
Supplement: Ostfeld et al. supplementary material 2 — Ostfeld et al. supplementary material [file S0031182024000349sup002.pdf]

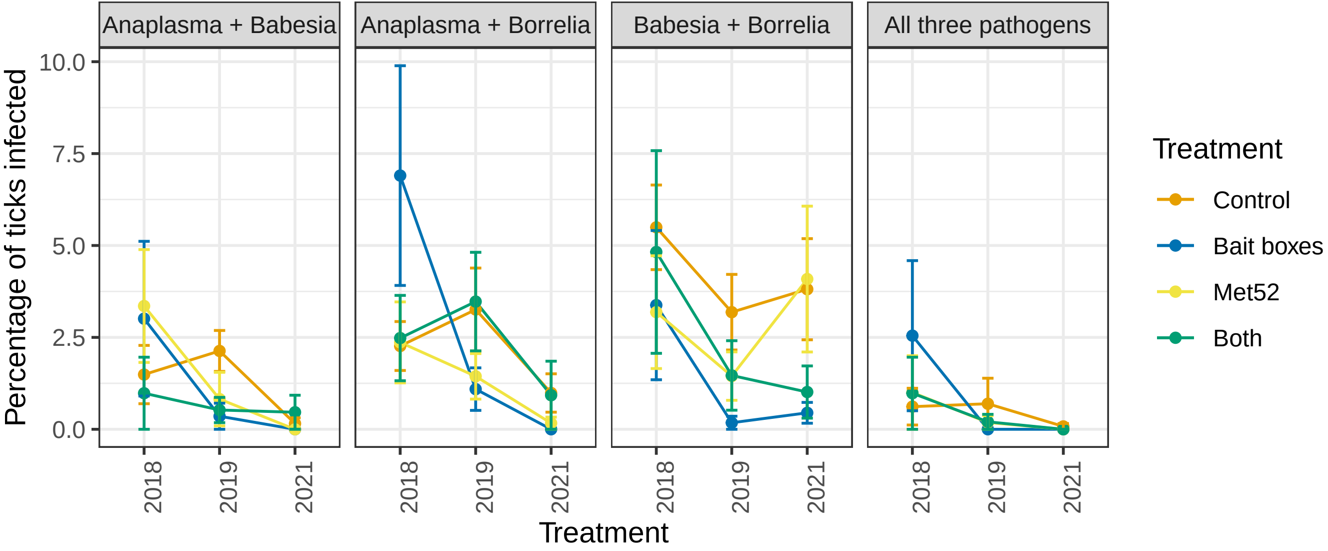

Anaplasma + Babesia
Anaplasma + Borrelia
Babesia + Borrelia
All three pathogens
Percentage of ticks infected
10.0
7.5
5.0
2.5
0.0
2018
2019
2021
Treatment
Treatment
Control
Bait boxes
Met52
Both
