# Supplementary material for: Effects of residential acaricide treatments on patterns of pathogen coinfection in blacklegged ticks
Source: Parasitology. 2024 Mar 18;151(9):946–52. doi: 10.1017/S0031182024000349 (PMC11770522; doi:10.1017/S0031182024000349)
Supplement: Ostfeld et al. supplementary material 5 — Ostfeld et al. supplementary material [file S0031182024000349sup005.docx]

**Table S1**. Predictions of infection prevalence in questing nymphal blacklegged ticks using a permutation analysis assuming independent assortment of all three pathogens, and deviations of observed data from those predictions. Values for ticks collected in neighbourhoods receiving one of four treatments in The Tick Project (controls, bait boxes, Met 52 spray, both bait boxes and Met52 spray) were calculated separately.

| **CONTROL NEIGHBORHOODS** | |  |  |  |  |  |
| --- | --- | --- | --- | --- | --- | --- |
| **Infection** | **Mean Expected Prevalance** | **2.5% quantile** | **97.5% quantile** | **Actual Prevalence** | **p-value** | **Observed:Expected** |
| Anaplasma | 10.53 | 9.77 | 11.30 | 11.30 | 0.054 | 1.07 |
| Babesia | 5.99 | 5.30 | 6.64 | 4.02 | **0.000** | 0.67 |
| Borrelia | 15.66 | 14.81 | 16.48 | 14.50 | **0.006** | 0.93 |
| Anaplasma + Babesia | 1.01 | 0.57 | 1.47 | 0.89 | 0.681 | 0.89 |
| Anaplasma + Borrelia | 2.64 | 1.98 | 3.32 | 1.72 | **0.010** | 0.65 |
| Babesia + Borrelia | 1.50 | 0.96 | 2.04 | 3.32 | **0.000** | 2.21 |
| All three pathogens | 0.25 | 0.06 | 0.51 | 0.51 | **0.045** | 2.02 |
| No pathogens | 62.43 | 61.49 | 63.35 | 63.73 | **0.006** | 1.02 |
|  |  |  |  |  |  |  |
| **NEIGHBORHOODS WITH BAIT BOXES** | |  |  |  |  |  |
| **Infection** | **Mean Expected Prevalance** | **2.5% quantile** | **97.5% quantile** | **Actual Prevalence** | **p-value** | **Observed:Expected** |
| Anaplasma | 8.10 | 7.20 | 9.08 | 8.07 | 1.000 | 1.00 |
| Babesia | 3.61 | 2.88 | 4.32 | 3.17 | 0.237 | 0.88 |
| Borrelia | 15.23 | 14.12 | 16.14 | 14.99 | 0.675 | 0.98 |
| Anaplasma + Babesia | 0.42 | 0.00 | 0.86 | 0.43 | 1.000 | 1.04 |
| Anaplasma + Borrelia | 1.76 | 1.01 | 2.59 | 1.59 | 0.742 | 0.90 |
| Babesia + Borrelia | 0.79 | 0.29 | 1.44 | 1.01 | 0.475 | 1.28 |
| All three pathogens | 0.09 | 0.00 | 0.29 | 0.29 | 0.128 | 3.19 |
| No pathogens | 70.01 | 69.02 | 71.18 | 70.46 | 0.435 | 1.01 |
|  |  |  |  |  |  |  |
| **NEIGHBORHOODS WITH MET52** | |  |  |  |  |  |
| **Infection** | **Mean Expected Prevalance** | **2.5% quantile** | **97.5% quantile** | **Actual Prevalence** | **p-value** | **Observed:Expected** |
| Anaplasma | 8.01 | 7.26 | 8.76 | 7.86 | 0.702 | 0.98 |
| Babesia | 6.13 | 5.37 | 6.77 | 3.98 | **0.000** | 0.65 |
| Borrelia | 11.77 | 10.95 | 12.54 | 10.15 | **0.000** | 0.86 |
| Anaplasma + Babesia | 0.69 | 0.30 | 1.19 | 0.90 | 0.410 | 1.29 |
| Anaplasma + Borrelia | 1.33 | 0.70 | 1.99 | 1.00 | 0.356 | 0.75 |
| Babesia + Borrelia | 1.02 | 0.50 | 1.59 | 2.69 | **0.000** | 2.64 |
| All three pathogens | 0.11 | 0.00 | 0.40 | 0.40 | **0.027** | 3.47 |
| No pathogens | 70.93 | 70.05 | 71.84 | 73.03 | **0.000** | 1.03 |
|  |  |  |  |  |  |  |
| **NEIGHBORHOODS WITH BOTH BAIT BOXES AND MET52** | | |  |  |  |  |
| **Infection** | **Mean Expected Prevalance** | **2.5% quantile** | **97.5% quantile** | **Actual Prevalence** | **p-value** | **Observed:Expected** |
| Anaplasma | 10.83 | 9.56 | 11.95 | 10.92 | 0.894 | 1.01 |
| Babesia | 5.77 | 4.78 | 6.83 | 4.95 | 0.139 | 0.86 |
| Borrelia | 13.96 | 12.63 | 15.19 | 12.29 | **0.013** | 0.88 |
| Anaplasma + Babesia | 0.96 | 0.34 | 1.71 | 0.34 | 0.106 | 0.35 |
| Anaplasma + Borrelia | 2.34 | 1.37 | 3.41 | 2.56 | 0.752 | 1.10 |
| Babesia + Borrelia | 1.24 | 0.51 | 2.05 | 2.39 | **0.008** | 1.92 |
| All three pathogens | 0.21 | 0.00 | 0.68 | 0.51 | 0.120 | 2.46 |
| No pathogens | 64.69 | 63.31 | 66.21 | 66.04 | 0.076 | 1.02 |
